# Experience of Human Papillomavirus Vaccination Project in a Community Set Up-An Indian Study

**DOI:** 10.31557/APJCP.2021.22.3.699

**Published:** 2021-03

**Authors:** Ranajit Mandal, Dipanwita Banerjee, Krishnendu Gupta, Puja Chatterjee, Manisha Vernekar, Chandrima Ray

**Affiliations:** 1 *Department of Gynaecological Oncology, Chittaranjan National Cancer Institute, 37 S P Mukherjee Road, Kolkata-700026, West Bengal, India. *; 2 *Department of Gynaecology and Obstetrics, Vivekananda Institution of Medical Sciences, 99 Sarat Bose Road, Kolkata-26, India. *; 3 *Department of Surgical Oncology, Ram Manohar Lohia Institute of Medical Sciences, Vibhuti Khand, Gomti Nagar, Lucknow, 226010, Uttar Pradesh, India. *

**Keywords:** HPV vaccine in India, vaccination in community setup, acceptability of HPV vaccine

## Abstract

**Background:**

Initial introduction of HPV vaccination from 2006 to 2008 was largely confined to high-income countries (HIC), such as Australia, the United States, and Europe, where cervical cancer incidence is lowest. Much of the post-introduction literature has come from HICs, with a focus on coverage levels achieved, provider acceptability and early impact of vaccination on disease endpoints. However, there are a few literature evaluating the mechanics of delivery, feasibility of the health system and acceptability from low and middle income countries (LMICs). The primary objective was to evaluate the feasibility, acceptability and safety of two dose HPV vaccination in adolescent girls between 9-14 years.

**Methods:**

After an orientation camp followed by filling up of prevaccine questionnaires by parents on HPV related diseases and its vaccines and informed consent, girls between9-14years were vaccinated. They were asked to report any side effects in the next 24 hours after each dose. Parents were contacted on Day 7 and Day 30 to enquire about any side effects . Total 3 visits were required i.e two for the vaccination and one visit at 7th month post completion of second dose. To estimate the acceptability, successful completion of two doses by at least 80% of the girls were measured. For measurement of acceptability, either of the parents were recalled along with their daughter at 7th month and were asked to fill up a pre-set questionnaire.

**Results:**

After institutional ethical clearance, 555 girls were recruited in the study from rural parts of West Bengal, India between July, 2017 to November, 2017. Out of which, 544 girls (98%) received their 2^nd^ dose between January, 2018 and May, 2018 without any serious adverse effects. No serious adverse effect was reported on follow up till December, 2019.

**Conclusion:**

The introduction of HPV vaccination is feasible in large scale and the vaccine is well accepted and safe.

## Introduction

Background: Since the introduction of cervical cancer screening by Papanicolaou smear from cervical cytology in 1940s, various methods have evolved with latest addition of Human papillomavirus Deoxyribonucleic acid (DNA) and/ Ribonucleic acid (RNA) test to detect the causative agent and treat accordingly. In India, current clinical guidelines recommend screening to start from 25years of age (Bhatla et al., 2019). If resources available, women aged 30 and older should be screened using various Polymerase Chain Reaction (PCR) based or hybridization technique based detection of HPV DNA (Abreu et al., 2012; Malloy et al., 2000; Nahvijou et al., 2014). These tests are also recommended for women of any age with abnormal cytology results. As primary prevention, the World Health Organization (WHO) recommends targeting HPV vaccination to girls prior to their sexual debut as it has been proven that the efficacy of vaccine is maximum when administered prior to exposure to the virus (Basu et al., 2013). Vaccination against Human papillomavirus (HPV) is an attractive way of cervix cancer prevention especially in resource constrained settings. Initial introduction of HPV vaccination from 2006 to 2008 was largely contained in high-income countries, such as Australia, the United States, and Europe, where the rates of cervical cancer are lowest. Much of the post-introduction literature has come from high-income countries, with a focus on coverage levels achieved, provider acceptability, and early impact of vaccination on disease endpoints (Malloy et al., 2000).However, there are very few literature evaluating the mechanics of delivery and feasibility of the health system to incorporate HPV vaccine in low and middle income countries (LMICs). Experiences from implementation in high-income countries may not necessarily be relevant or translatable to low-income countries, which have the most to gain from adoption of HPV vaccine, given that more than 80 percent of all deaths from cervical cancer occur in these countries (Nahvijou et al., 2014). Only few studies are available regarding acceptability of this vaccine in the community, feasibility in establishing a vaccine program in national scale and cost-effectiveness particularly in the Indian context (Paul et al.,2014; Kaarthigeyan, 2010). By establishing the evidences from pilot projects, HPV vaccination may therefore represent an efficiency saving, making programmes more sustainable within the context of a comprehensive approach to cervical cancer prevention and control as developed by WHO. The primary objective of this community based pilot project was to evaluate the feasibility, acceptability and safety of the two dose HPV vaccination in adolescent girls between 9-14 years. The secondary objective was to evaluate the awareness about the cervical cancer and its vaccine in the community.

## Materials and Methods

The Department of Gynaecological Oncology, Chittaranjan National Cancer Institute (CNCI), Kolkata,West Bengal conducted this project. The project was started in July, 2017 after obtaining institutional ethical clearance (IEC NO. A-4.311/03/2017). A large number of participants were from the rural areas of three nearby districts due to their proximity to CNCI. Well established network with the nongovernmental organisations (NGO), village heads, panchayat officers and other local stakeholders was convenient for us to reach out to the community without facing much difficulty. 

The vaccine used in this project was Gardasil^® ^manufactured by MSD India. As an act of benevolence, the vaccines were donated to CNCI by the Rotary Club of Calcutta Victoria, District 3291.CNCI is pioneer in conducting community based cervical cancer screening projects over last two decades. From 2017, an integrated project on screening for non communicable disease (IPNCD) is running at CNCI under the Department of Gynaecological Oncology. The project staffs involved in the project are well trained and have more than 10 years of experience in field work. The project is funded by the Ministry of Health and Family Welfare, Govt of India. The logistics related to the vaccine camp were utilised from the existing project of IPNCD. Before starting the project in community set-up, we had focus group discussions (FGD) with willing members of the civil society organizations especially in rural community.We also involved a few local clinicians to sensitize the NGO heads and local stake holders before developing the questionnaire. The team of Doctor, social worker and project co-ordinator from the Department visited these camps where the local authorities brought eligible girls with either of their parents from the local community and held an orientation cum awareness session on HPV related diseases and its vaccines. The local organizers played a key role to encourage the local community. Parents in different villages were approached through house to house visits and only willing parents were invited to take part in the project. This was a big limitation of our study as standardization was not possible among the data collectors. The demographic composition of the communities could not be calculated as there was no record of how many parents were approached and how many refused. We encouraged bringing both parents on the day of vaccination camp. The tentative attendance of the camp was calculated prior hand by requesting the local authority to do the registration process of the interested participants aged between 9-14 years in their locality at least 1 month prior to the scheduled camp. This was done to get a rough estimate of the willing participants so that approximate numbers of vaccines were carried with five extra doses on the vaccination day. Before the actual recruitment, an orientation talk was delivered by the Doctor about the actual process along with the eligibility criteria for the study. The inclusion criteria for recruitment were:

1. Girls aged between 9 to 14 years 2. Not suffering from any debilitating illness. 3.The girls sign the assent form and 4. No history of prior HPV vaccination and 5. Parents sign informed consent form

After exclusion of non eligible participants, the parents who agreed to take part in the study, a closed end survey questionnaire was distributed amongst either of the parents to test their knowledge of HPV vaccine,cervical cancer and HPV infection.

This was followed by vaccination of their daughters at free of charge on the same day. The Project Coordinator or the Social worker collected all survey forms for every camp. No leading questions were asked while filling up the forms. 

Recruitment was truly voluntary. Even after filling up the survey forms, the parents were free to withdraw their daughter from the project at any point of time. The eligible girls received the vaccine on their upper arm in sitting posture. After vaccination, girls were advised to wait for another 30 minutes before leaving for home. WHO recommended two dose schedules were administered at 0 and 6 month with all necessary precautions. An emergency tray containing lifesaving drugs were readily available with the team for treatment of anaphylactic shock if any. Some camps arranged refreshments for the participants at the end of vaccination procedure. The vaccines were carried in a vaccine carrier box by one of the designated person from the pharmaceutical company who used to reach the camp along with the team from CNCI thus maintaining the cold chain. The unused vaccines were taken back to CNCI and kept in the refrigerator between 2-8 degree at the end of the day until we used them in next camp. 

**Figure 1:**
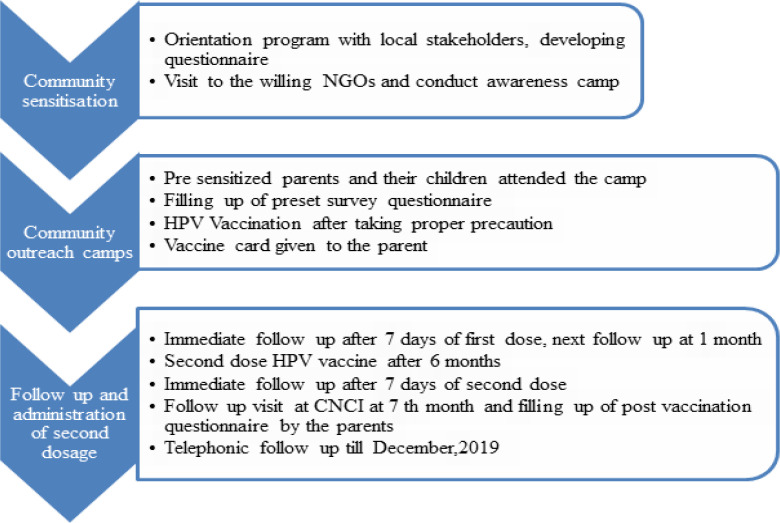
Study Algorithm

At the time of recruitment, all subjects received a vaccine card in their local language for documentation and were requested to carry the card during each visit. The respondents were asked to report any side effects over telephone or in person in the next 24 hours after each dose. Parents were contacted on Day 7 and Day 30 post vaccination to enquire about any side effects Apart from mild pain, no serious adverse effects were reported by the parents.Total 3 visits were required for the project i.e two for the vaccination and one visit at 7th month post completion of second dose. The study algorithm is mentioned in Figure 1.

To estimate the acceptability, successful completion of two doses by at least 80% of the girls were measured. Either of the parents were recalled along with their daughter at 7^th^ month and were asked to fill up a pre-set questionnaire to express their level of satisfaction and if they recommended the vaccine to any other parents in previous months. Total five girls who refused the second dose couldnot be contacted. We reviewed their initial filled up questionnaires and did not find any comment to indicate that they were unwilling to receive the first dose or had any intention not to complete the second dose. However they neither did any negative publicity about the vaccine nor approached to the other girls of the same locality who completed their second dose We could complete the project without any untoward event and were even approached by the local people to conduct the project at a large scale.

## Results

Total 8 camps were organized between July, 2017 to November, 2017 spreading across three districts of West Bengal. The number of girls attended these camps were 612, out of which 57 girls were not included in the project The major reason for exclusion were age >15 years, high fever on previous night and one girl had a history of epileptic attack in previous week.Total 555 girls between 9-14 years were recruited in the study, number of girls between 9-11 years and 12-14 years are 276 (49.7%) and 279 (50.3%) respectively. The socio-demographic characteristics are described in Table 1. Table 2 describes the adverse effects reported by the participants or their parents within 7 days of vaccination. Most common side effect reported was mild pain (N=126, 22.7% after first dose, N=120,22% after second dose).Other reported side effects were redness, swelling, nausea, fatigue mostly reflecting flu like symptoms. It was noted from the pre vaccination survey that 525 out of 555 parents (94.6%) were not aware of the HPV infection or its related vaccination and cervical cancer screening. However, in post vaccination survey, they expressed their satisfaction with the vaccine and did not express any negative comments in their form about the vaccine and its related side effects. The objective of inclusion of awareness about the HPV vaccine, cervical cancer and its screening amongst the parents was to assess their knowledge and attitude towards accepting a relatively new vaccine as a prevention of a disease like cancer. No serious adverse events including hospital admission were reported. Total 544 girls (98%) did receive their 2^nd^ dose between January, 2018 and May, 2018 without any serious adverse effects. Rest of 11 girls did not receive the second dose. The reason for their discontinuation were:

1. Three girls moved out from West Bengal along with their parents.

2. One had a fracture hip and was in hospital and did not turn up in future communications.

3. Two girls were unwell during their second dose schedule and,

4. Five girls were not willing to complete their second dose following their guardian’s advice. 

In view of WHO recommendation of HPV vaccine administration can be done even after 12-15 months of 1^st^ dose, we tried to contact the rest of the 11 girls whom we could not vaccinate at first hand. Rest 544 girls were carefully followed up over telephone till December, 2019 and were specifically asked for altered menses, any hospital admission for any medical event in last 1.5 years No significant side effects of the vaccines were reported.

**Table 1:** Sociodemographic Characteristics

Categories		Number
Age ( years)	9-11	276 (49.7%)
	12-14	279 ( 50.3%)
Religion	Hinduism	423 (76%)
	Islam	132 (24%)
Residence	Urban	12 (2%)
	Rural	543 (98%)
Menarche	Yes	148 (27%)
	No	407 (73%)
Education		
	Standard IV	16 (2.9%)
	Standard V	84 ( 15.1%)
	Standard VI	176 (31.7%)
	Standard VII	168 (30.3%)
	Standard VIII	110 (19.8%)
	Standard IX	1 (0.2%)
Language	Bengali	527 ( 94.9%)
	Hindi	28 (5.04%)

**Table 2:** Side Effects Reported within First Week of Each Vaccination Dose

Symptoms	First dose (N=555)	Second dose (N=544)
Redness	10 (1.8%)	12 (2.2%)
Swelling	15 (2.7%)	14 (2.52%)
Rash	0 (0%)	2 (0.36%)
Pain at local site	126 (22.7%)	120 (22%)
Temperature (>100^o^F)	0 (0%)	0 (0%)
Headache	15 (2.7%)	12 (2.2%)
Fatigue	12 (2.2%)	5 (0.91%)
GI Symptoms	10 (1.8%)	5 (0.91%)
Arthralgia	0 (0%)	0 (0%)
Myalgia	0 (0%)	0 (0%)

## Discussion

Since 2008, a handful of middle-income countries, such as Mexico and Panama, have phased in HPV vaccine with purchases at discounted prices through the Pan-American Health Organization (PAHO) Revolving Fund (Luciani et al., 2018). Globally three licensed HPV vaccines are available.Cervarix^®^(GlaxoSmithKline Biologicals) targets HPV genotypes 16 and 18; GARDASIL^®^ (Merck & Co. Inc.) targets HPV 16, 18, 6, 11; GARDASIL-9 (Merck & Co. Inc.) targets an additional five oncogenic genotypes 31, 33, 45, 52 and 58 (Zhai and Tumban, 2016) Between 2007 and 2012, several countries conducted HPV demonstration projects with vaccines provided by the GARDASIL^® ^Access Program (GAP), Merck & Co., the Bill & Melinda Gates Foundation through PATH, or through other means (Luciani et al., 2018; Zhai and Tumban, 2016; Gallagher et al., 2017).In 2012 GAVI, the Vaccine Alliance, commenced support for demonstration projects and national introductions to increase access to HPV vaccine worldwide (Ladner et al., 2016). It is unclear how much knowledge the general population in India has about HPV, such as how the virus is transmitted, how infection can be detected, whether it is linked to cervical cancer and availability of a vaccine. Several trials have been conducted in last few years Although, majority of the sample population in these trials are from urban background and no large community study has been done previously to check what is going on in the rural areas. The results of this study may help guide the design and improvement of interventions and campaigns to sensitize women to HPV infection and vaccination, thereby helping reduce incidence of cervical cancer. The results of our study are comparable to the high acceptance of HPV vaccine reported among parents in Mysore, India and also to the actual vaccine coverage rates reported in a vaccine delivery pilot project in Andhra Pradesh and Gujarat (Levinet al., 2014). The high acceptance rates of vaccination among survey population in Delhi may be due to the detailed information the participants received about cervical cancer and the vaccines. In Kolkata, Basu and Mittal (2011) had reported an increase in the acceptance level of vaccine after parents were provided with detailed factsheets about cervical cancer and HPV vaccines ( Jacob et al., 2010).

HPV vaccination programme targeted to adolescent girls provides a potential opportunity to build up immunogenicity against these two high risk oncogenic type of the HPV i.e. HPV 16 and HPV 18.Introduction of HPV vaccination in immunization program of the state in Punjab and Sikkim is a welcome move for future prospect of this vaccine in the country. Delhi Government also did an opportunistic vaccination with high coverage and safety in recent times (Sankaranarayanan et al.,2019; Sankaranarayanan et al., 2016; Prinja et aL., 2017). Prospects of the two dose schedule extendable to 18 years age, single dose schedule providing comparable protection like two dose , and future availability of an affordable Indian vaccine currently undergoing Phase III clinical trial shows promise for future widespread implementation and evaluation of HPV vaccination in India (Mehrotra et aL., 2018; Basu et al., 2016; Sankaranarayanan et al., 2018; Bhatla et al., 2018, Basu et al., 2019). Because HPV vaccines are targeted at girls who are not routinely receiving other vaccines or other public health interventions, establishing an HPV vaccination programme is more challenging and requires systems for social mobilization, cold chain and other logistics to establish school-based or outreach delivery. As with the successful components of implementation, the negative experiences and challenges are also helpful in starting a relatively new vaccination in a large populous country like India. Examples of HPV vaccine implementation within the routine immunization systems of Asian countries are largely absent. The study was able to deliver an insight to the current situation of HPV vaccination in a real life community set up. Our study had a few limitations. For example, the sample size of the study was small as we could get the vaccination for limited number. We chose the districts where we already had a well-established network with the local authorities thus required relatively less effort to recruit from the community. The same may have been difficult if we had chosen other districts far off from the institute. The distance from all the sites were within 100 kilometre from CNCI and were well connected with the city. Each outreach camps were in close proximity to a nearby hospital where 24X7 emergency facilities were available. A sense of comfort also worked at the back of our mind regarding management of un toward complication if there were any. The questionnaires developed were purely based on FGDs with involvement of local stake holders, NGO s and even local practicing clinicians. Open ended questions would have given a more appropriate reflection of the awareness and knowledge of the parents involved in the project. Parents of different villages covered by the NGOs were approached. Therefore the data on total number of parents approached, participants refused and also how many of the willing participants actually turned up on the day of vaccination were not measured. Involving children is always a sensitive issue. Measuring awareness in such a project where people who are concerned about their children’s health always creates a biased information. But the manuscript has described the experience of vaccinating adolescent girls in rural India in an opportunistic ‘campaign mode’. The high compliance to the second dose and no reported serious adverse effect of the vaccine reflects the acceptance of this vaccine even when not administered in a health care set up. The study has established that with increasing awareness and education, successful implementation of HPV vaccination is feasible, safe and well accepted in the community. Current cost of the vaccine is definitely brings a clear question on affordability, especially in India, therefore inclusion of this vaccine in immunization program of the country will be beneficial for the rural less affordable population of the country. 

The study reflects the real life scenario of a community based HPV vaccination program. A major milestone in cervical cancer prevention in some parts of the country has been the introduction of HPV vaccine as a state program for adolescent girls. Educating health-care professionals on how to communicate with parents to encourage HPV vaccination would greatly enhance the uptake. The introduction of HPV vaccination is feasible but there is an urgent need to sensitize the community about HPV and its related infections. The social media can play a significant role in mobilizing mass mindset and acceptance level of HPV vaccines. Lastly, as health is a state subject each state can include this vaccine in their annual health care budget to distribute it as a part of their immunization program till the time it is available through National Immunization program. 

## Author Contribution Statement

Study conception and design by DB; Data collection by DB, RM, KG, PC, MV, CR; Analysis and Interpretation of results by DB, KG, RM, MV; Draft Manuscript preparation by DB, RM, PC. All authors reviewed the results and approved the final version of the manuscript.
